# Autophagy-related gene LC3 expression in tumor and liver microenvironments significantly predicts recurrence of hepatocellular carcinoma after surgical resection

**DOI:** 10.1038/s41424-018-0033-4

**Published:** 2018-07-02

**Authors:** Chih-Wen Lin, Yaw-Sen Chen, Chih-Che Lin, Po-Huang Lee, Gin-Ho Lo, Chia-Chang Hsu, Pei-Min Hsieh, Kah Wee Koh, Tsung-Ching Chou, Chia-Yen Dai, Jee-Fu Huang, Wan-Long Chuang, Yao-Li Chen, Ming-Lung Yu

**Affiliations:** 1 Division of Gastroenterology and Hepatology, E-Da Dachang Hospital, I-Shou University, Kaohsiung, Taiwan; 2 Division of Gastroenterology and Hepatology, Department of Medicine, E-Da Hospital, I-Shou University, Kaohsiung, Taiwan; 3 Health Examination Center, E-Da Hospital, I-Shou University, Kaohsiung, Taiwan; 4 School of Medicine, College of Medicine, I-Shou University, Kaohsiung, Taiwan; 5 Department of Surgery, E-Da Hospital, I-Shou University, Kaohsiung, Taiwan; 6 Department of Surgery, Kaohsiung Chang Gung Memorial Hospital and Chang Gung University College of Medicine, Kaohsiung, Taiwan; 7 Department of Surgery, National Cheng-Kung University Hospital, Tainan, Taiwan; 8 Hepatobiliary Division, Department of Internal Medicine, and Hepatitis Center, Kaohsiung Medical University Hospital, and Center for Infectious Disease and Cancer Research, Kaohsiung Medical University, Kaohsiung, Taiwan; 9 Division of General Surgery, Department of Surgery, Changhua Christian Hospital, Changhua, Taiwan; 10 Institute of Biomedical Sciences, National Sun Yat-Sen University, Kaohsiung, Taiwan; 11 Center for Intelligent Drug Systems and Smart Bio-devices, College of Biological Science and Technology, National Chiao Tung University, Hsin-Chu, Taiwan

## Abstract

**Background:**

The role of autophagy-related markers as the prognostic factor of post-operative hepatocellular carcinoma (HCC) recurrence remained controversial.

**Methods:**

Overall, 535 consecutive HCC patients undergoing curative resection from 2010 to 2014 were followed and classified with early (ER, <2 years) or late recurrence (LR). Autophagy-related markers, LC3, Beclin-1, and p62 expression was immunohistochemically assessed in HCC and adjacent non-tumor (ANT) tissues.

**Results:**

HCC recurred in 245 patients: 116 with ER and 129 with LR. The cumulative incidence of recurrence at 1, 3, 5, and 7 years was 9.7%, 33.9%, 53.3%, and 66.3%, respectively. In multivariate analysis, HCC recurrence was significantly associated with low LC3 expression in tumor and ANT tissues, HCC tissues only and ANT tissues only (hazard ratio/95% confidence interval: 6.12/2.473–17.53, 4.18/1.285–13.61, and 1.89/1.299–2.757) and macrovascular invasion (1.63/1.043–2.492) and cirrhosis (1.59/1.088–2.326). ER was significantly associated with low LC3 expression in tumor and ANT tissues, HCC tissues only and ANT tissues only (6.54/2.934–15.81, 3.26/1.034–10.27, and 2.09/1.313–3.321) and macrovascular and microvascular invasion (2.65/1.306–5.343 and 2.55/1.177–5.504). LR was significantly associated with low LC3 expression in tumor and ANT tissues, HCC tissues only and ANT tissues only (5.02/1.372–18.83, 3.19/1.13–12.09, and 1.66/1.051–2.620) and cirrhosis (1.66/1.049–2.631). Patients with low and high LC3 expression in tumor and ANT tissues showed a 5-year cumulative recurrence of 94.3% and 41.7%, respectively (*p* < 0.001).

**Conclusions:**

The high LC3 expression in the tumor and liver microenvironments is significantly associated with lower HCC recurrence. Furthermore, tumor characteristics and liver microenvironment were also significantly associated with ER and LR, respectively.

**Translational impact:**

The analysis for LC3 expression in both the HCC and ANT tissues could identify patients at risk of HCC recurrence.

## Introduction

Hepatocellular carcinoma (HCC) is currently the fifth most common type of cancer and the third leading cause of cancer-related mortality worldwide^1–3^. In Taiwan, HCC is highly associated with viral- and alcoholic-associated cirrhosis^4^ and ranks as the second leading cause of cancer-related death^5^. Even after curative resection for HCC, the 5-year recurrence rate and survival rate remain as high as 60 and 50%, respectively^6, 7^. Identifying the factors associated with HCC recurrence after surgical resection could provide a promising strategy to improve the prognosis of HCC patients undergoing curative hepatectomy.

Autophagy is involved in the physiology and pathogenesis of human disease^8, 9^. Enhancement or inhibition of autophagy-related proteins has been reported to have therapeutic efficacy in cancer patients^10, 11^. However, studies of the involvement of autophagy in tumor recurrence have yielded controversial results^12–15^. In addition, the prognostic significance of autophagy-related markers such as LC3 and Beclin-1 in predicting the clinical outcome of HCC patients has been reported in previous studies^13–18^, but results have been conflicting due to the relatively limited case numbers^19^.

Recently, the molecular and histological changes that occur in the tumor microenvironment have become a main area of focus. The non-tumor liver microenvironment plays an important role in hepatocarcinogenesis^20–23^. The presence of autophagy in the non-tumor microenvironment was also found to promote tumor growth via the provision of nutrients^24^. These findings highlight the importance of studying both the tumor and non-tumor microenvironments to comprehensively understand the impact of autophagy in HCC development and progression. Hence, we conducted the current large-scale study to explore the impact of autophagy-related markers in both the tumor and adjacent non-tumor (ANT) microenvironments on HCC recurrence after surgical resection. Our results suggest that the low LC3 expression in both tumor and ANT microenvironments strongly predicts HCC recurrence in patients who have undergone curative resection.

## Materials and methods

### Patients and follow-up

This retrospective study included 535 consecutive, histologically proven HCC patients who underwent curative surgical resection between 2010 and 2014 at E-Da Hospital, I-Shou University, Kaohsiung, Southern Taiwan (*n* = 318) and Changhua Christian Hospital, Changhua, Central Taiwan (*n* = 217). All patients received regular follow-up every 3 months after surgery. The follow-up period was defined as the duration from the date of operation to the date of either death or the last follow-up. The last follow-up was on December 2016. Time to recurrence (TTR) was defined as the duration from the date of operation to the date of recurrence. Recurrent HCC was defined based on histological confirmation or highly elevated serum alpha-fetoprotein (AFP) in addition to diagnosis via at least two imaging methods according to the recommendations of the American Association for the Study of Liver Disease (AASLD)^25^. The patients were divided into four groups according to their TTR: patients experiencing recurrence within 2 years after operation (early recurrence group, ER, *n* = 116); patients experiencing recurrence 2–7 years after operation (late recurrence group, LR, *n* = 129); an all-patient recurrence group (AR, *n* = 245), consisting of both the ER and LR groups; and patients with no recurrence during the follow-up period after the first hepatectomy (non-recurrence group, NR, *n* = 290).

For the remaining materials and methods, please see Supporting information.

## Results

### Baseline demographic data

The demographic and clinicopathological factors of the 535 patients (73.1% male, mean age of 63 years) are shown in Table 1. Regarding the etiology of HCC, 46.7% of the patients had HBV, 28.4% had HCV, 3.9% had HBV/HCV co-infection, and 20.9% were not infected with HBV/HCV. One-third of the patients had liver cirrhosis, of which 22.8 and 9.5%, respectively, had a Child-Pugh score of A and B. One-tenth of the patients had an Edmondson-Steiner Grade of I–II, and approximately one-fifth of the patients had multiple tumors. Macrovascular and microvascular tumor invasion were observed in 20.7 and 46.0% of the patients, respectively. Regarding tumor stage, 16.4 and 36.1% of the patients were TNM stage III–IV and BCLC stage B-C, respectively. Regarding the expression of autophagy-related markers, 91.6% of the HCC tissues and 59.8% of the ANT tissues were high for LC3; 86.7% of the HCC tissues and 34.8% of the ANT tissues were high for Beclin-1; and 81.1% of the HCC tissues and 8.4% of the ANT tissues were high for p62.

**Table 1:** Basic demographic data and univariate analysis of recurrence in all patients

Characteristics	All patients (*n* = 535)	Without recurrence (*n* = 290)	With recurrence, all (*n* = 245)	*p*-value
*Gender*				
Female	144 (26.9)	69 (23.8)	75 (30.6)	0.076
Male	391 (73.1)	221 (76.2)	170 (69.4)	
Age (years)	63.1 ± 11.5	62.3 ± 12.1	64.1 ± 12.7	0.076
HTN	101 (18.9)	58 (20.0)	43 (17.6)	0.471
DM	59 (11.0)	35 (12.1)	24 (9.8)	0.403
Alcohol	129 (24.9)	67 (23.1)	62 (25.3)	0.553
Smoking	152 (28.4)	83 (28.6)	69 (28.2)	0.907
*HCC etiology*				
Non HBVHCV	112 (20.9)	60 (20.7)	52 (21.2)	0.826
HBV	250 (46.7)	132 (45.5)	118 (48.2)	
HCV	152 (28.4)	85 (29.3)	67 (27.3)	
HBV + HCV	21 (3.9)	13 (4.5)	8 (3.3)	
AST (IU/L)	55 ± 38	56 ± 41	54 ± 34	0.412
ALT (IU/L)	50 ± 39	53 ± 40	48 ± 37	0.108
Total bilirubin (mg/dl)	0.79 ± 0.34	0.78 ± 0.36	0.81 ± 0.33	0.235
Albumin (g/dl)	3.9 ± 0.4	3.8 ± 0.5	3.9 ± 0.4	0.192
Creatinine	1.0 ± 0.7	1.1 ± 0.8	1.1 ± 0.9	0.676
Platelet count (×10^3^/ml)	175 ± 71	171 ± 71	179 ± 72	0.254
INR	1.07 ± 0.10	1.07 ± 0.13	1.09 ± 0.14	0.091
AFP (ng/dl)	2797 ± 13215	2699 ± 11535	2913 ± 14985	0.856
ICG (%)	8.3 ± 5.3	7.8 ± 4.9	8.5 ± 5.8	0.466
*Liver cirrhosis*				
Negative	362 (67.7)	211 (72.8)	151 (61.6)	**0.006**
Positive	173 (32.3)	79 (27.2)	94 (38.4)	
Child-Pugh score A	122 (22.8)	63 (21.7)	59 (24.1)	
Child-Pugh score B	51 (9.5)	16 (5.5)	35 (14.3)	
*Antiviral therapy*				
Negative	185 (43.7)	92 (40.0)	93 (48.2)	0.091
Positive	238 (56.3)	138 (60.0)	100 (51.8)	
*Operative methods*				
Minor LR	412 (77.0)	223 (76.9)	189 (77.1)	0.946
Major LR	123 (23.0)	67 (23.1)	56 (22.9)	
*Operative margin (* *>* *1* *cm)*				
Negative	150 (28.0)	81 (27.9)	69 (28.2)	0.952
Positive	385 (72.0)	209 (72.1)	176 (71.8)	
*Edmondson-Steiner grades*				
I–II	51 (9.5)	34 (11.7)	17 (6.9)	0.060
III–IV	484 (90.5)	256 (88.3)	228 (93.1)	
*Macrovascular invasion*				
Negative	424 (79.3)	240 (82.8)	184 (75.1)	**0.030**
Positive	111 (20.7)	50 (17.2)	61 (24.9)	
*Microvascular invasion*				
Negative	289 (54.0)	158 (54.5)	131 (53.5)	0.815
Positive	246 (46.0)	132 (45.5)	114 (46.5)	
*Tumor number*				
Single	438 (81.9)	238 (82.1)	200 (81.6)	0.896
Multiple	97 (18.1)	52 (17.9)	45 (18.4)	
*Tumor size*				
<5 cm	352 (65.8)	191 (65.9)	161 (65.7)	0.971
≥5 cm	183 (34.2)	99 (34.1)	84 (34.3)	
*TNM stage*				
I–II	447 (83.6)	241 (83.1)	206 (84.1)	0.761
III–IV	88 (16.4)	49 (16.9)	39 (15.9)	
*BCLC stage*				
0-A	342 (63.9)	185 (63.8)	157 (64.1)	0.945
B-C	193 (36.1)	105 (36.2)	88 (35.9)	
*LC3 in tumor tissues*				
Low	45 (8.4)	11 (3.8)	34 (13.9)	**<.0001**
High	490 (91.6)	279 (96.2)	211 (86.1)	
*Beclin-1 in tumor tissues*				
Low	71 (13.3)	40 (13.8)	31 (12.7)	0.699
High	464 (86.7)	250 (86.2)	214 (87.3)	
*p62 in tumor tissues*				
Low	101 (18.9)	58 (20.0)	43 (17.6)	0.471
High	434 (81.1)	232 (80.0)	202 (82.4)	
*LC3 in ANT tissues*				
Low	215 (40.2)	93 (32.1)	122 (49.8)	**<.0001**
High	320 (59.8)	197 (67.9)	123 (50.2)	
*Beclin-1 in ANT tissues*				
Low	349 (65.2)	198 (68.3)	151 (61.6)	0.108
High	186 (34.8)	92 (31.7)	94 (38.4)	
*p62 in ANT tissues*				
Low	490 (91.6)	266 (91.7)	224 (91.4)	0.902
High	45 (8.4)	24 (8.3)	21 (8.6)	

Data shown as mean ± standard deviation or number (%). Patients with the presence of liver cirrhosis were further sub-classified as those with a Child-Pugh score of A and B. Patients infected with HBV and/or HCV were further classified as those with and without antiviral therapy

*HTN* Hypertension, *DM* Diabetes Mellitus, *HBV* Hepatitis B virus, *HCV* Hepatitis C virus, *AST* aspartate aminotransferase, *ALT* alanine aminotransferase, *INR* International normalized ratio, *AFP* Alpha-fetoprotein, *ICG* Indocyanine green, Minor liver resection: ≤2 segmentectomy, Major liver resection: ≥3 segmentectomy, *BCLC* stage Barcelona clinic liver cancer, *ANT* adjacent non-tumor

The significance of bold enteries in tables 1, 2 and 3 is p-value < 0.005.

**Fig. 1:**
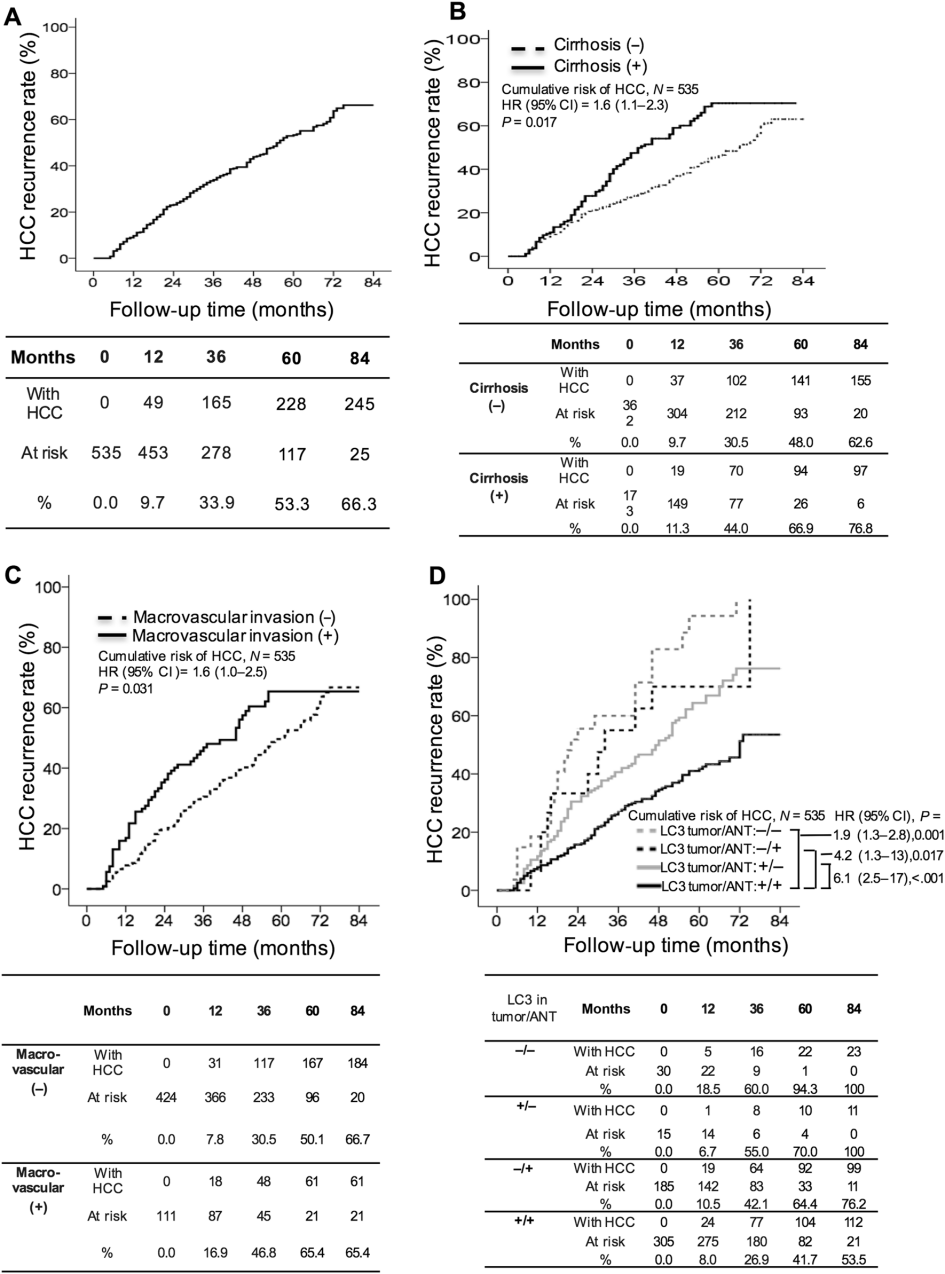
Cumulative incidence of HCC recurrence with respect to various clinicopathological factors. The cumulative incidence of HCC in all patients (**a**). Patients with the presence of liver cirrhosis (**b**) and macrovascular invasion (**c**) were significantly more likely to develop HCC recurrence. Patients low LC3 expression in the adjacent non-tumor (ANT) tissues (+/−), HCC tissues (−/+) or both (−/−) had a significantly higher incidence of recurrence than patients with LC3 expression in both HCC and ANT tissues (+/+; **d**). HCC hepatocellular carcinoma, ANT adjacent non-tumor, HR hazard ratio, CI confidence intervals, + high, − low

During the median follow-up of 42 months (range, 1–84 months), 245 patients experienced HCC recurrence, including 116 cases of ER and 129 cases of LR (incidence rate, 14.4% per person-year). Forty-two (17.1%) and 203 (82.9%) patients experienced extrahepatic recurrence and intrahepatic recurrence, respectively. The cumulative incidence of HCC recurrence at 1, 3, 5, and 7 years after HCC resection was 9.7%, 33.9%, 53.3%, and 66.3%, respectively (Fig. 1a).

### Factors related to HCC recurrence in patients who underwent hepatectomy

In univariate analysis, the presence of liver cirrhosis and macrovascular invasion and the low LC3 expression in HCC tissues or ANT tissues were significantly associated with HCC recurrence (Table 1).

In multivariate analysis, the Cox proportional hazard model identified that patients with low LC3 expression in both the HCC and ANT tissues had the highest risk of HCC recurrence (−/−; hazard ratio [HR]: 6.12; 95% confidence interval [CI]: 2.473–17.53), followed by those low LC3 expression in HCC tissues only (−/+; HR: 4.18; 95% CI: 1.285–13.61), those low LC3 in ANT tissues only (+/−; HR: 1.89; 95% CI: 1.299–2.757), those with macrovascular invasion (HR: 1.63; 95% CI: 1.043–2.492) and those with the presence of liver cirrhosis (HR: 1.59; CI: 1.088–2.326) (Table 3).

The 1-, 3-, 5-, and 7-year cumulative incidence of HCC recurrence was 11.3%, 44.0%, 66.9%, and 76.8%, respectively, in patients with liver cirrhosis and 16.9%, 46.8%, 65.4%, and 65.4%, respectively, in patients with macrovascular invasion, which were significantly higher levels than found in their counterparts (Fig. 1b, c, respectively). Next, LC3 expression was analyzed in parallel in HCC and ANT tissues. The results showed that patients with high LC3 expression in both tissues (+/+) had a 1-, 3-, 5-, and 7-year cumulative incidence of HCC recurrence of 8.0%, 26.9%, 41.7%, and 53.5%, respectively. Compared to this group, patients with low LC3 expression in both tissues (−/−; 18.5, 60.0, 94.3, and 100%, respectively), those low LC3 expression in HCC tissues only (−/+; 10.5%, 42.1%, 64.4%, and 76.2%, respectively) and those low LC3 expression in ANT tissues only (+/−; 6.7%, 55.0%, 70.0%, and 100%, respectively) were significantly more prone to HCC recurrence (Fig. 1d).

### Factors related to early HCC recurrence in patients who underwent hepatectomy

To identify factors associated with early HCC recurrence, clinicopathological features were compared between the ER group (*n* = 116) and patients with no recurrence within 24 months after operation (*n* = 419) (Table 2). The presence of macrovascular and microvascular invasion, tumor size ≥5 cm, advanced BCLC stage and low LC3 expression in HCC tissues or ANT tissues were significantly associated with the risk of early HCC recurrence.

**Table 2:** Univariate analyses of early and late recurrences

	Early recurrence			Late recurrence		
Characteristics	Without (*n* = 419)	With (*n* = 116)	*p*-value	Without (*n* = 290)	With (*n* = 129)	*p*-value
*Gender*						
Female	107 (25.5)	37 (31.9)	0.172	69 (23.8)	38 (29.5)	0.220
Male	312 (74.5)	79 (68.1)		221 (76.2)	91 (70.5)	
Age (years)	62.7 ± 11.9	64.3 ± 9.8	0.072	62.3 ± 12.1	63.7 ± 11.7	0.242
HTN	75 (17.9)	26 (22.4)	0.272	58 (20.0)	17 (13.2)	0.093
DM	47 (11.2)	12 (10.3)	0.791	35 (12.1)	12 (9.3)	0.407
Alcohol	99 (23.6)	30 (25.9)	0.619	67 (23.1)	32 (24.8)	0.705
Smoking	122 (29.1)	30 (25.9)	0.492	83 (28.6)	39 (30.2)	0.737
*HCC etiology*						
Non HBVHCV	85 (20.3)	27 (23.3)	0.268	60 (20.7)	25 (19.4)	0.601
HBV	189 (45.1)	61 (52.6)		132 (45.5)	57 (44.2)	
HCV	129 (30.8)	23 (19.8)		85 (29.3)	44 (34.1)	
HBV + HCV	16 (3.8)	5 (4.3)		13 (4.5)	3 (2.3)	
AST (IU/L)	56 ± 41	52 ± 34	0.372	56 ± 41	55 ± 37	0.704
ALT (IU/L)	53 ± 40	48 ± 38	0.056	53 ± 40	55 ± 42	0.970
Total bilirubin (mg/dl)	0.80 ± 0.35	0.79 ± 0.31	0.807	0.78 ± 0.36	0.84 ± 0.34	0.110
Albumin (g/dl)	3.8 ± 0.5	3.9 ± 0.4	0.349	3.8 ± 0.5	3.9 ± 0.4	0.332
Creatinine	1.1 ± 0.7	1.1 ± 0.9	0.275	1.1 ± 0.8	1.0 ± 0.8	0.815
Platelet count (×10^3^/ml)	173 ± 68	180 ± 59	0.351	171 ± 71	175 ± 71	0.492
INR	1.08 ± 0.14	1.07 ± 0.10	0.618	1.07 ± 0.13	1.10 ± 0.15	0.083
AFP (ng/dl)	2625 ± 9736	2223 ± 10381	0.598	2699 ± 11535	3533 ± 16514	0.604
ICG (%)	8.0 ± 4.8	8.6 ± 5.9	0.686	8.2 ± 5.2	9.1 ± 7.8	0.372
*Liver cirrhosis*						
Negative	291 (69.5)	71 (61.2)	0.093	212 (73.1)	79 (61.2)	**0.015**
Positive	128 (30.5)	45 (38.8)		78 (26.9)	50 (30.2)	
*Antiviral therapy*						
Negative	141 (42.2)	44 (49.4)	0.222	108 (49.5)	42 (40.4)	0.123
Positive	193 (57.8)	45 (50.6)		110 (50.5)	62 (59.6)	
*Operative methods*						
Minor LR	320 (76.4)	92 (79.3)	0.506	223 (76.9)	97 (75.2)	0.705
Major LR	99 (23.6)	24 (20.7)		67 (23.1)	32 (24.8)	
*Operative margin (* *>* *1* *cm)*						
Negative	122 (29.1)	28 (24.1)	0.291	81 (27.9)	41 (31.8)	0.423
Positive	297 (70.9)	88 (75.9)		209 (72.1)	88 (68.2)	
*Edmondson-Steiner grades*						
I–II	45 (10.7)	6 (5.2)	0.0721	34 (11.7)	11 (8.5)	0.329
III–IV	374 (89.3)	110 (94.8)		256 (88.3)	118 (91.5)	
*Macrovascular invasion*						
Negative	346 (82.6)	78 (67.2)	**<.0001**	240 (82.8)	106 (82.2)	0.884
Positive	73 (17.4)	38 (32.8)		50 (17.2)	23 (17.8)	
*Microvascular invasion*						
Negative	242 (57.8)	47 (40.5)	**0.001**	158 (54.5)	84 (65.1)	**0.042**
Positive	177 (42.2)	69 (59.5)		132 (45.5)	45 (34.9)	
*Tumor number*						
Single	339 (80.9)	99 (85.3)	0.272	238 (82.1)	101 (78.3)	0.364
Multiple	80 (19.1)	17 (14.7)		52 (17.9)	28 (21.7)	
*Tumor size*						
<5 cm	286 (68.3)	66 (56.9)	**0.022**	191 (65.9)	95 (73.6)	0.114
≥5 cm	133 (31.7)	50 (43.1)		99 (34.1)	34 (26.4)	
*TNM stage*						
I–II	353 (84.2)	94 (81.0)	0.409	241 (83.1)	112 (86.8)	0.884
III–IV	66 (15.8)	22 (19.0)		49 (16.9)	17 (13.2)	
*BCLC stage*						
0-A	280 (66.8)	62 (53.4)	**0.008**	185 (63.8)	95 (73.6)	**0.048**
B-C	139 (33.2)	54 (46.6)		105 (36.2)	34 (26.4)	
*LC3 in tumor tissues*						
Low	25 (6.0)	20 (17.2)	**<.0001**	11 (3.8)	14 (10.9)	**0.005**
High	394 (94.0)	96 (82.8)		279 (96.2)	115 (89.1)	
*Beclin-1 in tumor tissues*						
Low	55 (13.1)	16 (13.8)	0.851	40 (13.8)	15 (11.6)	0.545
High	364 (86.9)	100 (86.2)		250 (86.2)	114 (88.4)	
*p62 in tumor tissues*						
Low	79 (18.9)	22 (19.0)	0.978	58 (20.0)	21 (16.3)	0.369
High	340 (81.1)	94 (81.0)		232 (80.0)	108 (83.7)	
*LC3 in ANT tissues*						
Low	151 (36.0)	64 (55.2)	**<.0001**	95 (32.8)	56 (43.4)	**0.036**
High	268 (64.0)	52 (44.8)		195 (67.2)	73 (56.6)	
*Beclin-1 in ANT tissues*						
Low	260 (62.1)	65 (56.0)	0.067	186 (64.1)	74 (57.4)	0.075
High	159 (37.9)	51 (44.0)		104 (35.9)	55 (42.6)	
*p62 in ANT tissues*						
Low	381 (90.9)	109 (94.0)	0.297	266 (91.7)	115 (89.1)	0.397
High	38 (9.1)	7 (6.0)		24 (8.3)	14 (10.9)	

Data shown as mean ± standard deviation or number (%). Patients in the antiviral therapy group were those infected with HBV and/or HCV

*HTN* Hypertension, *DM* Diabetes Mellitus, *HBV* Hepatitis B virus, *HCV* Hepatitis C virus, *AST* aspartate aminotransferase, *ALT* alanine aminotransferase, *INR* International normalized ratio, *AFP* Alpha-fetoprotein, *ICG* Indocyanine green, Minor liver resection: ≤2 segmentectomy, Major liver resection: ≥3 segmentectomy, *BCLC* stage Barcelona clinic liver cancer, *ANT* adjacent non-tumor

The significance of bold enteries in tables 1, 2 and 3 is p-value < 0.005.

In multivariate analysis, the Cox proportional hazard analysis identified that patients with low LC3 expression in both HCC and ANT tissues had the highest risk of HCC recurrence (−/−; HR: 6.54; 95% CI: 2.934–15.81), followed by those low LC3 expression in HCC tissues only (−/+; HR: 3.26; 95% CI: 1.034–10.27), those with the presence of macrovascular and microvascular invasion (HR: 2.65; 95% CI: 1.306–5.343 and HR: 2.55; 95% CI: 1.177–5.504, respectively) and those low LC3 expression in ANT tissues only (+/−; HR: 2.09; 95% CI: 1.313–3.321) (Table 3).

**Table 3:** Multivariate analyses of factors associated with all recurrence, early recurrence, and late recurrence

Variables	Hazard ratio	95% CI	*p*-value
All recurrence			
*Liver cirrhosis*			
Negative	1		
Positive	1.59	1.088–2.326	**0.017**
*Macrovascular invasion*			
Negative	1		
Positive	1.63	1.043–2.492	**0.031**
*LC3 in tumor/ANT tissues*			
+/+	1		
+/−	1.89	1.299–2.757	**0.001**
−/+	4.18	1.285–13.61	**0.017**
−/−	6.12	2.473–17.53	**<.0001**
Early recurrence			
*Macrovascular invasion*			
Negative	1		
Positive	2.65	1.306–5.343	**0.017**
*Microvascular invasion*			
Negative	1		
Positive	2.55	1.177–5.504	**0.018**
*Tumor size*			
<5 cm	1		
≥5 cm	0.76	0.383–1.528	0.448
*BCLC stage*			
0-A	1		
B-C	0.52	0.214–1.240	0.139
*LC3 in tumor/ANT tissues*			
+/+	1		
+/−	2.09	1.313–3.321	**0.002**
−/+	3.26	1.034–10.27	**0.044**
−/−	6.54	2.934–15.81	**<.0001**
Late recurrence			
*Liver cirrhosis*			
Negative	1		
Positive	1.66	1.049–2.631	**0.031**
*Microvascular invasion*			
Negative	1		
Positive	0.78	0.361–1.699	0.537
*BCLC stage*			
0-A	1		
B-C	0.74	0.324–1.680	0.468
*LC3 in tumor/ANT tissues*			
+/+	1		
+/−	1.66	1.051–2.620	**0.030**
−/+	3.19	1.13–12.09	**0.021**
−/−	5.02	1.372–18.83	**0.011**

*ANT* adjacent non-tumor, *BCLC*stage Barcelona clinic liver cancer, + high, − low

The significance of bold enteries in tables 1, 2 and 3 is p-value < 0.005.

**Fig. 2:**
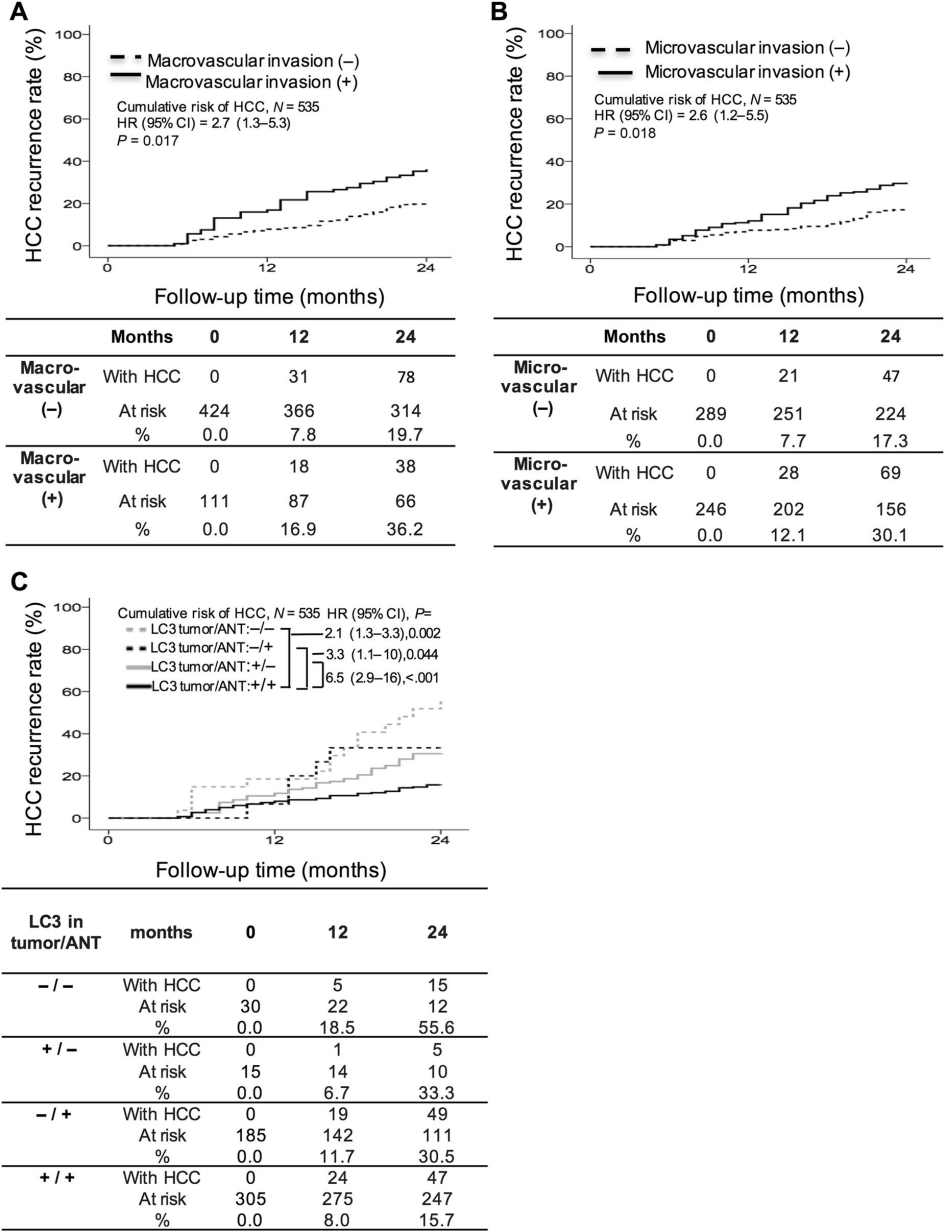
Cumulative incidence of early recurrence with respect to various clinicopathological factors. Patients with macrovascular invasion (**a**) and microvascular invasion (**b**) were significantly more likely to develop early recurrence of HCC. Patients low LC3 expression in HCC tissues (−/+), adjacent non-tumor (ANT) tissues (+/−), or both (−/−) had a significantly higher incidence of early recurrence than patients with LC3 expression in both HCC and ANT tissues (+/+; **c**). HCC hepatocellular carcinoma, ANT adjacent non-tumor, HR hazard ratio, CI confidence intervals, + high, − low

The 1- and 2-year cumulative incidence of HCC recurrence was 16.9% and 36.2%, respectively, in those with macrovascular invasion (Fig. 2a) and 12.1% and 30.1%, respectively, in those with microvascular invasion (Fig. 2b), which were significantly higher than those in their counterparts. To evaluate whether LC3 expression in HCC and ANT tissues has an effect on ER, LC expression patterns were analyzed in both tissue types. The results revealed a 1- and 2-year cumulative incidence of HCC recurrence of 8.0% and 15.7%, respectively, for patients with high LC3 expression in both tumor and ANT tissues (+/+). Compared to this group, patients with low LC3 in both tissues (−/−; 18.5% and 55.6%, respectively), those low LC3 in HCC tissues only (−/+; 11.7% and 30.5%, respectively) and those low LC3 in ANT tissues only (+/−; 6.7% and 33.3%, respectively) were significantly more prone to early recurrence (Fig. 2c).

### Factors related to late HCC recurrence in patients who underwent hepatectomy

To identify factors associated with late HCC recurrence, clinicopathological factors were compared between the LR (*n* = 129) and the NR groups (*n* = 290) (Table 2). In univariate analysis, the presence of liver cirrhosis and microvascular invasion, early BCLC stage, and the low LC3 expression in HCC tissues or ANT tissues were significantly associated with late HCC recurrence.

In multivariate analysis (Table 3), the Cox proportional hazard model identified that patients with low LC3 expression in both HCC and ANT tissues had the highest risk of HCC recurrence (−/−; HR: 5.02; 95% CI: 1.372–18.83), followed by those low LC3 expression in HCC tissues only (−/+; HR: 3.19; 95% CI: 1.13–12.09), those low LC3 expression in ANT tissues only (+/−; HR: 1.66; 95% CI: 1.051–2.620) and those with the presence of liver cirrhosis (HR: 1.66; 95% CI: 1.049–2.631).

**Fig. 3:**
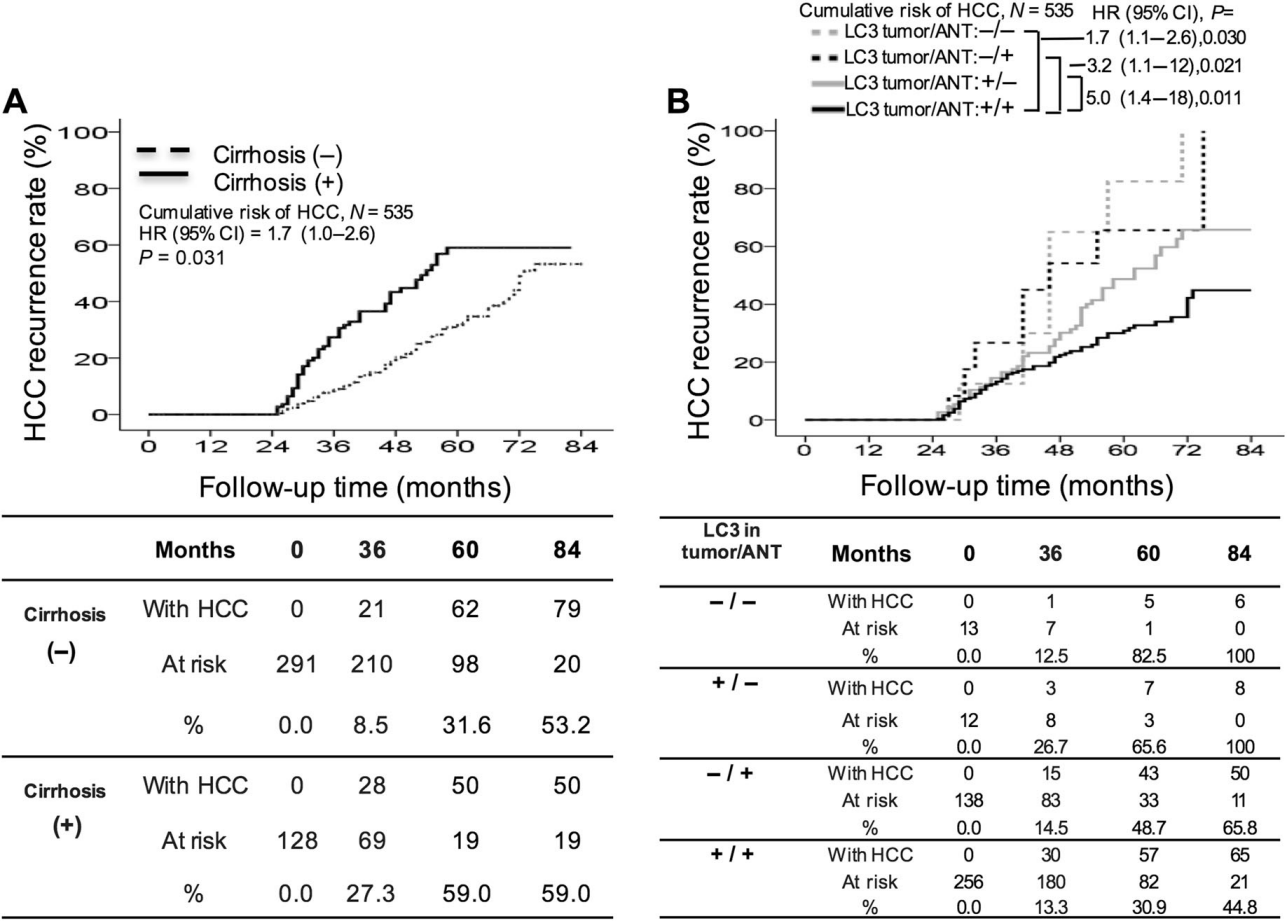
Cumulative incidence of late recurrence with respect to various clinicopathological factors. Patients with the presence of liver cirrhosis (**a**) were significantly more likely to develop late recurrence of HCC. Patients low LC3 expression in HCC tissues (−/+), adjacent non-tumor (ANT) tissues (+/−), or both (−/−) had a higher incidence of late recurrence than patients with LC3 expression in both HCC and ANT tissues (+/+; **b**). HCC hepatocellular carcinoma, ANT adjacent non-tumor, HR hazard ratio, CI confidence intervals, + high, − low

The 3-, 5-, and 7-year cumulative incidence of HCC recurrence was 27.3%, 59.0%, and 59.0%, respectively, for patients with liver cirrhosis, which was significantly higher than for those without liver cirrhosis (Fig. 3a). When LC3 expression was analyzed in parallel in tumor and ANT tissues, the 3-, 5-, and 7-year cumulative incidence of HCC recurrence was found to be 13.3%, 30.9%, and 44.8%, respectively, for patients with LC3 expression in both tissues (+/+). Compared to this group, patients with low LC3 expression in both tissues (−/−; 12.5%, 82.5%, and 100%, respectively), those low LC3 expression in HCC tissues only (−/+; 14.5%, 48.7%, and 65.8%, respectively) and those low LC3 expression in ANT tissues only (+/−; 26.7%, 65.6%, and 100%, respectively) were significantly more prone to late HCC recurrence (Fig. 3b).

### LC3 staining of tumor and non-tumor tissues in re-operated patients

Having established the association between LC3 expression and HCC recurrence in patients who underwent first curative HCC hepatectomy, we next evaluated LC3 expression patterns in patients who underwent second (*n* = 32) and third (*n* = 5) operable HCC hepatectomy due to HCC recurrence (Table 4). The low LC3 expression in both tumor and ANT tissues was significantly associated with repeated HCC recurrence. Four and 3 patients showed a change from high to low LC3 staining in tumor tissues at the second and third surgical resection, respectively. Moreover, 5 and 4 patients showed a change from high to low LC3 staining in ANT tissues at the second and third surgical resection, respectively. In total, 100% of the patients showing a loss-of-LC3 staining in tumor and ANT tissues experienced HCC recurrence. This result further demonstrates that loss-of-LC3 expression in both HCC and ANT tissues is associated with a high risk of HCC recurrence, highlighting that LC3 expression significantly predicts HCC recurrence.

**Table 4:** LC3 staining of the tumor and adjacent non-tumor tissues in 32 re-resected patients

Characteristics	First resection (*n* = 32)	*p*-value	Second resection (*n* = 32)	*p*-value	Third resection (*n* = 5)	*p*-value
*LC3 in tumor tissues*						
Low	25 (78.1)	<0.001	29 (90.6)	<0.001	5 (100)	<0.001
High	7 (21.9)		3 (9.4)		0 (0)	
*LC3 in ANT tissues*						
Low	22 (68.8)	<0.001	27 (84.4)	<0.001	4 (80)	<0.001
High	10 (31.2)		5 (15.6)		1 (20)	

*ANT* adjacent non-tumor

## Discussion

Our study demonstrated that the high LC3 expression in both the tumor and non-tumor liver microenvironments is significantly associated with lower recurrence, regardless of early or late recurrence. This suggests that the measurement of LC3 expression in both tissues may serve as a predictor of HCC recurrence. The findings that the majority of the patients who underwent second and third operable hepatectomy also had a low LC3 expression in both tumor and ANT tissues and that the loss-of-LC3 expression in both HCC and ANT tissues led to a high risk of HCC recurrence further support the use of LC3 as a prognostic factor for HCC recurrence. In addition to LC3, vascular invasion was significantly associated with ER, and the presence of liver cirrhosis was significantly associated with LR. These results suggest that tumor characteristics have a higher impact on ER, whereas the liver microenvironment has a higher impact on LR.

Tumor risk factors such as tumor invasion into the portal vein and intrahepatic metastasis have been reported to be associated with ER^26^. In the current study, patients with vascular tumor invasion were at a significantly higher risk of developing ER. We postulate that oncogenes and tumor suppressor genes may have undergone genetic alterations during tumor progression^27^, hence allowing tumor cells to acquire invasive and metastatic potential. Intrahepatic metastasis might have occurred prior to hepatectomy or during tumor manipulation, contributing to ER^26, 27^. Cirrhosis is associated with carcinogenic potential and is also a predisposing factor for HCC recurrence^28^. Patients with pre-existing cirrhosis have been reported to have lower rates of recurrence-free survival at 3 years or later, suggesting that underlying liver status has an effect on LR^29^. This finding supports our observation that patients with liver cirrhosis, which denotes poor liver function, are more susceptible to LR. According to our results, the presence of vascular tumor invasion and liver cirrhosis may serve as prognostic factors for ER and LR, respectively.

Using a *Drosophila melanogaster* malignant tumor model, Katheder et al.^24^ recently demonstrated that dormant autophagy-deficient and growth-impaired tumors are capable of reactivating tumor growth when transplanted into an autophagy-efficient host, suggesting that autophagy in the microenvironment impacts tumor growth. Although many studies have revealed that LC3 expression in HCC tissues is higher than in non-tumor tissues and have associated LC3 expression in HCC tissues with tumor development and prognosis^14, 18, 30^, the relationship between LC3 expression in the non-tumor microenvironment and HCC progression has not been discussed in the literature. Here, we showed that the high LC3 expression in the tumor and ANT microenvironments have additional protective effects against HCC recurrence. Our results clearly demonstrate the importance and potential role of LC3 expression in the tumor and non-tumor liver microenvironments in the prognosis of HCC recurrence. This study is the first to demonstrate that LC3 expression in the non-tumor liver microenvironment is significantly associated with HCC recurrence and that the low LC3 expression in both tumor and ANT tissues significantly increases the risk of HCC recurrence.

Our findings indicate that patients with low LC3 expression in both tumor and ANT tissues are significantly more likely to experience first and second HCC recurrence. All the patients with a loss-of-LC3 staining in tumor and ANT tissues experienced recurrent HCC. The loss-of-LC3 in tumor and ANT tissues was also associated with a high risk of HCC recurrence. Regardless of any other HCC tumor characteristics or the status of ANT tissues in the liver, a low LC3 expression in tumor and ANT tissues at the time of surgery was associated with a significantly increased risk of HCC recurrence. This finding suggests that autophagy-related marker LC3 predicts HCC recurrence.

Our results revealed that the high LC3 in both the tumor and liver microenvironments provides patients who undergo curative hepatectomy with a survival advantage against HCC recurrence. However, there are some limitations to the current study. First, this study used a retrospective design, which could have resulted in unintended bias. Second, the findings of this study must still be validated in Western populations with different ethnicities. Third, the involvement of LC3 expression in the tumor and non-tumor microenvironment in limiting tumorigenesis related to HCC recurrence and its underlying mechanism related to HCC need further investigation in vivo and in vitro.

In summary, the low LC3 expression in both the tumor and non-tumor liver microenvironments was significantly associated with a very high risk of HCC recurrence in patients who underwent curative hepatectomy for HCC. Different factors were associated with early and late recurrence: while the presence of vascular tumor invasion was associated with ER, the liver microenvironment was associated with LR. This study is the first to demonstrate that, in addition to the tumor microenvironment, assessment of autophagy-related markers in the non-tumor liver microenvironment is very important for predicting HCC recurrence. The analysis of LC3 expression in tumor and ANT tissues, in conjunction with an assessment of the presence of vascular tumor invasion and liver cirrhosis, could identify patients at risk of HCC recurrence after curative resection. Our results indicated that autophagy-related marker LC3 is significantly associated with HCC recurrence and that LC3 may serve as a potential biomarker for predicting HCC recurrence.

## Study highlights

### What is current knowledge

- Autophagy is involved in the physiology and pathogenesis of human disease, including HCC.
- Autophagy-related markers, such as LC3 and Beclin-1 are used as prognostic factors of HCC.
- The impact of autophagy-related markers on post-operative HCC recurrence is not documented.

### What is new here

- LC3 expression in the tumor and liver microenvironments is associated with risk of post-operative HCC recurrence.
- The tumor characteristics and liver microenvironments have effects on early and late recurrence, respectively.

### Translational impact

- LC3 may serve as a potential biomarker for predicting post-operative HCC recurrence.

## Electronic supplementary material

Supplementary Materials and methods
