# Supplementary material for: Autophagy-related gene LC3 expression in tumor and liver microenvironments significantly predicts recurrence of hepatocellular carcinoma after surgical resection
Source: Clin Transl Gastroenterol. 2018 Jul 2;9(6):166. doi: 10.1038/s41424-018-0033-4 (PMC6026596; doi:10.1038/s41424-018-0033-4)
Supplement: Supplementary file 1 — Supplementary Materials and methods [file 41424_2018_33_MOESM1_ESM.docx]

**Supplemental Materials**

**MATERIALS AND METHODS**

**Patients and follow-up**

This retrospective study included 535 consecutive, histologically-proven HCC patients who underwent curative surgical resection between 2010 and 2014 at E-Da Hospital, I-Shou University, Kaohsiung, Southern Taiwan (n = 318) and Changhua Christian Hospital, Changhua, Central Taiwan (n = 217). All patients received regular follow-up every three months after surgery. The follow-up period was defined as the duration from the date of operation to the date of either death or the last follow-up. The last follow-up was December 2016. Time to recurrence (TTR) was defined as the duration from the date of operation to the date of recurrence. Recurrent HCC was defined based on histological confirmation or highly elevated serum alpha-fetoprotein (AFP) in addition to diagnosis via at least two imaging methods according to the recommendations of the American Association for the Study of Liver Disease (AASLD).^1^ The patients were divided into four groups according to their TTR: patients experiencing recurrence within 2 years after operation (early-recurrence group, ER, n = 116); patients experiencing recurrence 2-7 years after operation (late-recurrence group, LR, n = 129); an all-patient recurrence group (AR, n=245), consisting of both the ER and LR groups; and patients with no recurrence during the follow-up period after the first hepatectomy (non-recurrence group, NR, n = 290).

Clinicopathological parameters, including demographic data, hepatitis markers and serum AFP levels, were examined. The pathological stages of HCC were established using the American Joint Committee on Cancer TNM staging system^2^ and Barcelona Clinic Liver Cancer (BCLC) staging system.^3^ For pre-operative risk assessment, ICG-R15 was conducted to check for operable status.^4^ The functional status of the liver was evaluated using the Child-Pugh scoring system,^5^ and tumor histological grading was performed using the Edmondson–Steiner grading system.^6^ Among the included patients, 77.0% underwent minor liver resection (≤ 2 segmentectomy), and 23% underwent major liver resection consisting of either 3-4 segmentectomy (11.8%) or > 4 segmentectomy (11.2%). Both the HCC and ANT tissues that were between 0.5 – 5 cm from the negative operative margin were collected and stored in 4% paraformaldehyde until required. Patients with HBV and/or HCV infections were treated with antiviral therapy according to Taiwan Association for the Study of the Liver. Of the 250 and 152 patients with HBV and HCV infection, 163 and 57 patients were treated, respectively, with nucleoside/nucleotide analogs and pegylated interferon with ribavirin or direct-acting antiviral agents achieving sustained virologic response. Of the 21 patients with HBV/HCV co-infection, 18 patients were treated with nucleoside/nucleotide analogs, pegylated interferon with ribavirin or direct-acting antiviral agents. The study was conducted in accordance with the guidelines of the International Conference on Harmonization for Good Clinical Practice and was approved by the Ethics Committee of each participating hospital. All of the participants provided informed consent.

**Tissue microarray construction**

Tissue microarrays were constructed according to manufacturer instructions (Array Biotechnology Co., Taiwan). Briefly, all HCC specimens were stained with hematoxylin and eosin (H&E) and examined by two histopathologists. Representative areas free from necrotic and hemorrhagic materials were marked in paraffin blocks. Two cylindrical tissue cores (1.6-mm diameter) were removed from the donor blocks and transferred to recipient paraffin blocks, and their planar array positions were noted. Each contained about 96 cylinders, and the final tissue microarrays consisted of 535 HCC tissue samples along with the corresponding ANT tissues. Consecutive sections (4-μm thick) were cut from the array blocks and placed on adhesion microscope slides for immunohistochemical staining.

**Immunohistochemical staining and scoring**

The 4-μm tissue sections were stained using the HRP (DAB) detection system according to the manufacturer’s instructions with slight modifications. The following primary polyclonal antibodies were used: anti-LC3 (NB100-2220, Novus Biologicals, Littleton CO, USA), anti-Beclin-1 (ab51031, Abcam, Cambridge, UK), and anti-p62 (H00008878-M01, Abnova, Taipei, Taiwan). The semi-quantitative Immunoreactive-Score (IRS) system described previously was employed to semi-quantitate the expression of autophagy-related markers (LC3, Beclin-1, and p62; Supplemental Figure S1).^7^ The immunostaining scores were calculated according to the intensity and percentage of positive staining of all slides in this study. The intensity score was defined as 0 (no staining), 1 (weak staining), 2 (moderate staining) and 3 (strong staining). The percentage score was defined as 0 (None, no staining), 1 (< 10% positivity), 2 (10–50%) and 3 (≥ 50%) (Supplemental Figure S1). The intensity score and percentage score were multiplied together to obtain a total score. The expression was defined as either low (IRS <2) or high (IRS ≥ 2) based on the products of the intensity and percentage scores. All the slides were evaluated independently in a blinded manner by two investigators. Cases with discrepancies were discussed with other pathologists until a consensus was reached to confirm the score.

**Data analysis and statistics**

Data management and statistical analyses were performed using SPSS ver. 18.0 (SPSS, Chicago, IL, USA). Associations between each marker and clinical characteristics were evaluated using Pearson’s χ2 test and Fisher’s exact test. Correlation coefficients between the expression level of each marker were determined using Spearman’s correlation analysis. To evaluate whether the variables selected in the univariate analysis were independent factors for tumor recurrence, multivariate analyses was performed using a Cox’s proportional hazard regression model. Dummy variables were generated to sort variables into mutually exclusive categories for multiple regression analysis. The cumulative incidence of HCC recurrence was calculated using the Kaplan–Meier method and compared using the log-rank test. All statistical analyses were two-sided, and a *p*-value <0.05 was considered significant.

**REFERENCES**

1. Bruix J, Sherman M, Practice Guidelines Committee AAftSoLD. Management of hepatocellular carcinoma. Hepatology 2005;42:1208-36.

2. Edge SB, Compton CC. The American Joint Committee on Cancer: the 7th edition of the AJCC cancer staging manual and the future of TNM. Ann Surg Oncol 2010;17:1471-4.

3. Llovet JM, Bru C, Bruix J. Prognosis of hepatocellular carcinoma: the BCLC staging classification. Semin Liver Dis 1999;19:329-38.

4. de Liguori Carino N, O'Reilly DA, Dajani K*, et al.* Perioperative use of the LiMON method of indocyanine green elimination measurement for the prediction and early detection of post-hepatectomy liver failure. Eur J Surg Oncol 2009;35:957-62.

5. Pugh RNH, Murray-Lyon IM, Dawson JL*, et al.* Transection of the oesophagus for bleeding oesophageal varices. British Journal of Surgery 1973;60:646-649.

6. Edmondson HA, Steiner PE. Primary carcinoma of the liver: a study of 100 cases among 48,900 necropsies. Cancer 1954;7:462-503.

7. Lin CW, Lin CC, Lee PH*, et al.* The autophagy marker LC3 strongly predicts immediate mortality after surgical resection for hepatocellular carcinoma. Oncotarget 2017;8:91902-91913.
